# Development and Implementation of an Influenza Point-Of-Care Testing Service in a Chain Community Pharmacy Setting

**DOI:** 10.3390/pharmacy8040182

**Published:** 2020-10-06

**Authors:** Rachel Hardin, Pheli Roberts, Brooke Hudspeth, Angela Tracy, Lauren Baldwin, Michael Raque, Clark D. Kebodeaux

**Affiliations:** 1 Kroger Louisville Division, University of Kentucky College of Pharmacy, Louisville, KY 40223, USA; rachel.hardin@stores.kroger.com (R.H.); pheli.roberts@kroger.com (P.R.); angela.tracy@kroger.com (A.T.); lauren.baldwin@stores.kroger.com (L.B.); michael.raque@stores.kroger.com (M.R.); 2 Pharmacy Practice and Science, University of Kentucky College of Pharmacy, Lexington, KY 40509, USA; brooke.hudspeth@uky.edu

**Keywords:** point-of-care testing, community pharmacy, influenza

## Abstract

Point-of-care testing is becoming increasingly commonplace in community pharmacy settings. These tests are often used in the management of chronic disease, such as blood sugar, hemoglobin A1c and lipid levels, but can also be used for acute conditions such as influenza infection and group A streptococcus pharyngitis. When used for these acute infections, point-of-care tests can allow for pharmacist-initiated treatment. In this study, an influenza point-of-care testing service was developed and implemented in a chain community pharmacy setting and a retrospective review was conducted to assess the service. Of patients tested, 29% tested positive for influenza A and/or B; 92% of patients testing positive received a prescription as a result. While health insurance cannot be billed for the service due to current pharmacy reimbursement practices, this did not appear to negatively affect patient willingness to participate. As point-of-care testing services become more commonplace in community pharmacy settings, patient awareness will similarly increase and allow for more widespread access to acute outpatient care.

## 1. Introduction

There are an increasing number of opportunities for pharmacists to provide direct patient care services as the profession of pharmacy evolves. A significant source of these opportunities is the use of point-of-care (POC) tests. POC tests are performed outside of a laboratory and are waived under the Clinical Laboratory Improvement Amendments of 1988 (CLIA) [1]. The tests produce rapid and reliable results that can be administered in a variety of health care settings to allow for screening or monitoring of disease.

Some CLIA-waived tests are already commonly used in community pharmacies. Pharmacists provide screening for blood glucose and glycated hemoglobin, lipid panels, and even international normalized ratio (INR) in some settings. POC tests can also be used to test for infectious diseases such as influenza, HIV, adenovirus, and hepatitis C [2].

POC tests can be used in tandem with collaborative care agreements (CCAs) and board-of-pharmacy approved protocols to allow for pharmacist-initiated medication dispensing and/or professional services. In Kentucky, the legislation allows for the establishment of protocols for a variety of conditions [3]. To date, 13 protocols have been approved by the Kentucky Board of Pharmacy, including tobacco cessation, tuberculin skin testing, opioid use disorder, and acute uncomplicated urinary tract infection treatment among others [4].

Three of the 13 protocols utilize point-of-care (POC) testing results to guide medication selection and dispensing for treatment of infectious diseases. The current protocols allow pharmacists to use POC tests to test for acute influenza infection, acute uncomplicated urinary tract infection, and acute group A streptococcal pharyngitis infection. Subsequently, eligible patients may be treated appropriately pursuant to the outlined treatment plans.

Unique models such as these, especially those regarding pharmacy-based influenza management, have been in the works for some time, both in jurisdictions around the United States and in various other countries [5]. Studies have been conducted to evaluate the effectiveness of similar influenza and streptococcal programs to quantify the value and positive outcomes in other states for pharmacy-based POC implementation [6,7].

These models can greatly improve infection control, especially for seasonal influenza. The World Health Organization (WHO) estimates that there are three to five million cases of severe illness and about 290,000–650,000 respiratory deaths due to influenza worldwide each year [8]. For an illness where early detection and medication initiation are crucial to increase medication effectiveness, testing and treatment services offered by pharmacists—long-heralded as the most accessible healthcare provider—can bring significant value to the healthcare system as more pharmacies adopt this practice. In fact, one POC testing study found that nearly 40% of patients were seen at the pharmacy outside of “normal” physician office hours, highlighting the importance of increased testing convenience to ensure appropriate treatment [9].

While these testing services have the potential to improve patient access and the POC tests utilized in these services must be simple and have a low risk of incorrect result, errors may still occur in the testing process [10]. Manufacturer’s instructions must be followed to the letter and testing personnel must be trained on the equipment and testing procedure. Without proper training, these tests, and subsequently these services, have the potential for negative health outcomes if performed incorrectly.

The aim of this study is to describe the development and implementation of an influenza POC testing service in a large community pharmacy chain, identify successes and barriers to the implementation, and identify areas of improvement to enhance POC testing services in a community pharmacy setting.

## 2. Materials and Methods 

An influenza POC testing service was developed for a large community pharmacy chain. Development of the service was a multifaceted process. First, pertinent state laws and regulations as well as the board of pharmacy and company policies were reviewed to ensure uniform compliance across the varied regional divisions. Individual state requirements within the United States for protocols and collaborative care agreements may differ greatly; in these instances, it must be decided if the company policy will simply follow the more stringent rule, or if there will be slightly different policies where these distinctions arise. In general, state-based protocols are more restrictive and provide very specific guidelines to be followed whereas CCAs can be drafted to allow for greater use of a pharmacist’s clinical judgment.

Additionally, current clinical services offered across the pharmacy chain, such as naloxone dispensing and tobacco cessation counseling, were reviewed. These served as rough models for workflow processes and templates for required documentation forms, when appropriate.

Next, the company’s unified protocol had to be established. Screening criteria such as patient eligibility and inclusion and exclusion criteria were established. These were evaluated and pooled predominately from state existing state protocols and included age, vital signs, duration of symptoms, and other clinical factors. Prescribers with which collaborative relationships existed were identified and approached to gauge willingness to serve as the protocol prescriber. Due to different state requirements, different practitioners were used in the two different regional divisions.

Core paperwork was created. These were generated with the overall workflow in mind to simplify the process for the pharmacists providing the service. A patient questionnaire, treatment algorithm, visit summary, primary care provider notification sheet, and work/school excuse were all needed. A process guide was made to outline the various steps of the service, including which staff member (i.e., pharmacist or technician) would complete each step, core paperwork to be completed in each step, and the necessary documentation accompanying it.

Pharmacist training also had to be created. Training had to be robust and include topics such as an influenza disease state and treatment overview, patient eligibility, pharmacist responsibilities under the protocol/CCA, and counseling requirements. Pharmacists were also trained on performing the Rapid Influenza Diagnostic Test (RIDT), including patient evaluation, proper specimen collection, and machine use and maintenance. All pharmacy staff were trained on the service workflow as well as documentation requirements. Some states have specific training requirements built into protocols and collaborative care agreements and therefore some aspects of training differed across the pharmacy chain; the associated training organization was not consistent.

Finally, assurance measures were developed and put in place for ongoing service evaluation. These measures included regular meetings of the implementation team to review service data and were used to identify further areas of improvement such as additional staff training, altered workflow responsibilities, and altered documentation practices. The quality assurance measures also provide a structural platform for the addition of new clinical services as the profession expands further.

The service was then implemented in two regional divisions of the pharmacy chain. Patients presenting to the pharmacy with flu-like symptoms were evaluated by a trained pharmacist using the influenza service algorithm and pharmacist clinical judgment. Evaluation included measuring vital signs, assessing signs/symptoms, and obtaining a relevant medical history. If appropriate, patients were tested using a CLIA-waived RIDT. Pharmacists were then able to dispense an antiviral medication to patients testing positive for Influenza A and/or B, pursuant to the protocol/CCA. Counseling was provided and covered influenza vaccination, infection control measures, and appropriate self-care. Patients were referred for urgent or emergency care when necessary. Pharmacist follow-up occurred 24–72 h later via telephone.

A retrospective review was conducted to assess the influenza POC testing service. Prior to review, the study was approved by the University of Kentucky Institutional Review Board. Records from patient encounters occurring from 26 January 2020 to 2 March 2020 were reviewed. Data collected included date of service, patient gender, influenza vaccine status, blood pressure, heart rate, temperature, RIDT results (including both Influenza A and B), if the patient was referred or a prescriber was consulted during the visit, and if the patient received a prescription medication as a result of the visit. Additional information, such as patient symptoms, aggravating or alleviating factors, and time since symptom onset, was available for some patients. Data were analyzed using descriptive statistics.

## 3. Results

42 patients were provided the influenza testing service at 31 different locations. All 42 patients were included in the analysis. Documentation was available for all patients who completed screening. Patient information can be found in Table 1. Additional information regarding patients who tested positive for Influenza A and/or B can be found in Table 2.

For patients testing positive, treatment options were outlined in the service protocol and included oseltamivir, baloxavir marboxil or zanamivir. Antiviral agent selection was based on patient-and medication-specific factors as well as insurance formulary preference and cost. Specific agents to patients testing positive was not collected. A total of 11 of 12 patients received a prescription based on their RIDT result; the reason for one patient not receiving therapy was not recorded.

Of the 31 locations that provided the service, two locations provided the service to three patients, seven locations provided the service to two patients, and the rest of the locations provided the service to one patient over the 37-day study period.

Distribution of POC tests administered were evenly spread across days of the week. 71% of tests performed occurred on traditional business days (9.5% of visits occurred on a Monday, 19% on a Tuesday, 7.2% on a Wednesday, 14.3% on a Thursday, 19% on a Friday) and 31% of visits occurred on the weekend (12% on a Saturday, and 19% of visits on a Sunday).

Patient-reported symptoms were available for 27 of the patients. The most commonly-reported symptoms included fever, myalgia, and cough. Full symptom information can be found in Table 3.

## 4. Discussion

These services continue to shape the practice of community pharmacy. While POC testing services are not yet ubiquitous in pharmacies, the potential impact that they could have on patient accessibility and public health if they were to become a routine part of practice cannot be ignored. This regional implementation of an influenza related protocol shows the potential value that widespread implementation of POC influenza testing in community pharmacies can have on the healthcare system. Despite the initial implementation of the service over the 2019–2020 influenza season, the service was provided equally across the implementation pharmacies and not driven by a single location.

There are significant physician shortages in the United States, both current and projected [11]. The primary care physician shortage is especially noteworthy, with a projected 21,100 to 55,200 shortage by 2032. Currently, retail clinics (typically staffed by nurse practitioners) can serve as an alternative to traditional primary care providers and fill some of the void caused by physician shortages. Community pharmacies already aid in this role as well by performing services such as medication therapy management, immunization administration, health screenings, and disease prevention [12]. Given that around 90% of the US population lives within five miles of a community pharmacy, POC testing by pharmacists would be a convenient addition to pharmacy services, increase patient access to care, and help alleviate some of the strain on the primary care physician supply.

In many ways, the potential impact of increased presence of POC testing services can be compared to pharmacist-administered vaccines. Traditionally, immunizations were administered in physician clinics or primary care offices, public health systems such as community health clinics, or in hospitals [13]. Since the late 1990s and early 2000s, pharmacies have become another commonplace site for immunization administration. With the addition of pharmacists as immunizers, multiple studies have shown an increase in vaccinations provided, particularly in medically underserved areas [14]. Similarly, the accessibility of pharmacies, especially in medically underserved areas and for hard-to-reach populations, could greatly improve patient access to care via POC testing services.

POC testing services could also allow community pharmacies to serve as an even more valuable emergency response resource. Pharmacist roles are vast during an emergency or natural disaster, ranging from triaging patients and taking medication histories to administering vaccinations and providing emergency dispensing of necessary medications [15]. The many roles undertaken in emergency situations all fall within the pharmacist scope of practice and can be done in the absence of other healthcare providers. This allows additional practitioners to perform other duties to maximize the efficiency of the healthcare team.

For instance, pharmacists in the United States were authorized to order and administer COVID-19 tests. The influenza testing service had to be halted during the COVID-19 pandemic, however, because it was still in its infancy and the pharmacies did not have the capabilities or resources to facilitate coronavirus testing. Off-site testing facilities were commonly utilized, with pharmacists working alongside nurse practitioners and other providers to screen patients and administer tests. As POC testing services become more widespread across the country, pharmacies offering these services would be better primed to offer on-site testing, increasing access and availability as is needed in public health crises.

The implementation of such services is not without challenges. Protocols and CCAs must be approved and signed by a prescriber, defined as any individual authorized to prescribe a legend drug. Hesitant or unwilling prescribers could be a barrier to these collaborative programs, especially if practitioners are not aware of the current opportunities in this field [16]. However, one study found that physicians are especially open to collaborative practice agreements when there is already an established relationship with a community pharmacist [17]. The cultivation of such relationships is therefore crucial to the success of widespread service implementation.

Additionally, health insurance cannot be billed for the testing service due to current pharmacy reimbursement practices. In the United States, pharmacists are not recognized as providers on a national level, which leads to pharmacists being unable to bill patients’ health insurance for providing these and other services in the outpatient setting [18]. Although this could serve as a limitation in some areas, this did not appear to negatively impact patient willingness to participate in the service. Patients were willing to pay for the service out-of-pocket, indicating the usefulness of pharmacy-based POC testing despite the lack of insurance reimbursement. In countries where pharmacist provider status is not a concern, these services could be provided at a small or nonexistent out-of-pocket cost for the patient, further increasing convenience, patient access to care, and health outcomes. Furthermore, if and when pharmacists become recognized nationally as valued providers of care in the US, these improvements to patient health would similarly be seen.

Limitations of the retrospective review of a newly implemented POC include an unknown number of patients who may have approached the service but were not tested. All patients who completed the screening criteria were tested so it is unknown how many, if any, were screened but did not qualify for testing. Specific prescription data was not collected in aggregate since insurance coverage determined antiviral therapy if multiple options were available to patients. The implementation window for analysis did not span the entire 2019–2020 influenza season due to necessary implementation steps to meet required regulations. It is unknown if additional patients would have participated in the POC service if available from the onset of influenza season. Pharmacist training was individualized to each implementation site; a more standardized approach could better prepare pharmacists to offer the service and therefore increase patient safety. Prevalence of influenza activity in a given season can vary by state, and/or region, and can begin as early as November. Time of day was not measured, so it is unknown whether a higher percentage of visits occurred outside of normal physician office hours. However, while the exact time of implementation was not available, nearly 30% of the testing provided fell outside of traditional business days where patient may find care more difficult to access.

A noteworthy direction for future research is evaluation of the role that nearby or onsite retail clinics. Some large pharmacy chains have onsite clinics that offer similar services which could impact patient utilization of pharmacy POC services. Other next steps could include the implementation of an acute influenza infection chemoprophylaxis protocol which could provide appropriate dispensing of antiviral therapy for individuals at high risk (age 65 and older, chronic pulmonary disease, immunosuppression) when a patient tests positive for influenza. Another area for further investigation is time of day that these services occur. This study did not track the time at which patients presented to the pharmacy. Evaluation of peak hours of service utilization could assist in staff scheduling and other ways to optimize service efficiency and improve patient and pharmacist satisfaction.

## 5. Conclusions

Implementing protocol-driven influenza services can have a significant impact in community pharmacy settings. Patients were willing to pay for the service out-of-pocket, showcasing the need for pharmacy-based POC testing. There are different laws and regulations regarding these pharmacy services, so those must be compared and evaluated in order to implement a service in a specific area. As these services become more widespread, patient awareness increases, and the utility can be showcased on a larger scale, POC testing will continue to see growth in community pharmacy settings.

## Figures and Tables

**Table 1:** Patient information. RIDT: Rapid Influenza Diagnostic Test.

Category	# Of Patients (%)
**Gender**	
Male	20 (47.6)
Female	22 (52.4)
**RIDT Results**	
Positive	12 (28.6)
Negative	30 (71.4)
**Had Patient Received Flu Vaccine This Season?**	
Yes	22 (52.4)
No	20 (47.6)

**Table 2:** Patients with positive RIDT results.

Category	# (%)
**Influenza Type**	
A	9 (75)
B	2 (16.7)
Both A and B	1 (8.3)
**Did Patient Receive Prescription Based on RIDT?**	
Yes	11 (91.7)
No	1 (8.3)
**Had Patient Received Flu Vaccine This Season?**	
Yes	2 (16.7)
No	10 (83.3)

**Table 3:** Patient-reported symptoms.

Symptom	# of Patients (%)
Fever	16 (59.3)
Muscle ache	16 (59.3)
Cough	14 (51.9)
Headache	12 (44.4)
Malaise	10 (37)
Sore throat	8 (29.6)
Chills	7 (25.9)
Rhinitis	3 (11.1)
Chest tightness	2 (7.4)
